# The preliminary results of proton and carbon ion therapy for chordoma and chondrosarcoma of the skull base and cervical spine

**DOI:** 10.1186/s13014-019-1407-9

**Published:** 2019-11-21

**Authors:** Xiyin Guan, Jing Gao, Jiyi Hu, Weixu Hu, Jing Yang, Xianxin Qiu, Chaosu Hu, Lin Kong, Jiade J. Lu

**Affiliations:** 1 Department of Radiation Oncology, Shanghai Proton and Heavy Ion Center, 4365 Kangxin Road, Shanghai, 201315 China; 2 Department of Radiation Oncology, Shanghai Engineering Research Center of Proton and Heavy Ion Radiation Therapy, 4365 Kangxin Road, Shanghai, 201315 China; 3 Department of Radiation Oncology, Shanghai Proton and Heavy Ion Center, Fudan University Cancer Hospital, 4365 Kangxin Road, Shanghai, 201315 China

**Keywords:** Chordoma, Chondrosarcoma, Proton, Carbon ion, Radiotherapy

## Abstract

**Purpose:**

To evaluate the short-term outcomes in terms of tumor control and toxicity of patients with skull base or cervical spine chordoma and chondrosarcoma treated with intensity-modulated proton or carbon-ion radiation therapy.

**Methods:**

Between 6/2014 and 7/2018, a total of 91 patients were treated in our Center. The median age was 38 (range, 4–70) years. Forty-six (50.5%) patients were treated definitively for their conditions as initial diagnosis, 45 (49.5%) patients had recurrent tumors including 14 had prior radiotherapy. The median gross tumor volume was 37.0 (range, 1.6–231.7) cc. Eight patients received proton therapy alone, 28 patients received combined proton and carbon ion therapy, 55 patients received carbon-ion therapy alone.

**Results:**

With a median follow-up time of 28 (range, 8–59) months, the 2-year local control (LC), progression free (PFS) and overall survival (OS) rates was 86.2, 76.8, and 87.2%, respectively. Those rates for patients received definitive proton or carbon-ion therapy were 86.7, 82.8, and 93.8%, respectively. On multivariate analyses, tumor volume of > 60 cc was the only significant factor for predicting PFS (*p* = 0.045), while re-irradiation (*p* = 0.012) and tumor volume (> vs < 60 cc) (*p* = 0.005) were significant prognosticators for OS. Grade 1–2 late toxicities were observed in 11 patients, and one patient developed Grade 3 acute mucositis.

**Conclusions:**

Larger tumor volume and re-irradiation were related to inferior survival for this group of patients. Further follow-up is needed for long-term efficacy and safety.

## Introduction

Chordomas originate from transformed undifferentiated notochordal remnants that exist throughout the skull base and axial skeleton. The incidence of clival chordomas is approximately 0.8–1/10^6^ [1, 2]. Chondrosarcomas arise from cartilage and represent 10–20% of malignant bone tumors with 5–12% occurring in the head and neck, mostly at the skull base [3].

Both chordomas and chondrosarcomas are locally invasive, and surgery is their mainstay treatment. However, complete resection is nearly universally constrained for the skull base lesions by their proximity to critical neural or vascular structures [4]. Adjuvant radiation therapy (RT) can improve local control (LC) and overall survival (OS). A systematic review reported that the 5-year mortality rate decreased from 25 to 9% with the addition of any form of RT [3]. Nevertheless, high dose RT is usually not feasible due to the dose constrain of the critical organs at risk (OARs) particularly the optic nerve/chiasm, brain stem, spinal cord, and brain, if conventional photon-based RT technology is used. As such, long-term LC and OS remained suboptimal with the addition of adjuvant RT despite of the relatively indolent behavior of the conditions.

Proton and carbon ion beams have the physical advantage of a finite range in depth with a steep dose fall-off after the Bragg peak. In addition, compared to photon and proton beams, relative biological effectiveness (RBE) calculation results of about 3 for carbon ion beams within the target volume,which may theoretically has the enhanced advantage of biological advantages [5]. The effectiveness of particle radiation therapy for both chordoma and chondrosarcoma have been reported [4, 6]. Furthermore, dosimetry studies has confirmed that the use intensity-modulated proton (IMPT) and carbon ion radiotherapy (IMCT) with pencil beam scanning (PBS) technology could improve target volume coverage while minimizing the dose to the surrounding OARs thereby improving the therapeutic ratio for base of skull tumors [7].

In this paper, we try to bolster the literature with the results of a group of patients with skull base/cervical spine chordoma and chondrosarcoma treated with IMPT and IMCT at the Shanghai Proton and Heavy Ion Center (SPHIC).

## Patients and methods

### Pretreatment evaluation

Patient evaluation included a complete history and physical examination (H&P), complete blood count and electrolyte panel, renal and liver function tests, magnetic resonance imaging (MRI) of the head and neck region (CT was allowed when MRI was contraindicated), positron emission tomography (PET-CT) when appropriate. All cases were discussed in our institutional multidisciplinary tumor clinic for their indication for particle RT prior to registration and inclusion to the tumor registry.

### Immobilization and registration

All patients were registered and immobilized in the supine position with individualized thermoplastic masks. Planning CT scans without intravenous contrast from the vertex to the inferior margin of the clavicular heads were performed at 1 mm slice thickness. MRI-CT fusion was performed for all patients prior to target volume delineation. The gross tumor volume (GTV) consisted of the gross tumor discovered on clinical examination or imaging studies. We defined CTV-boost as a GTV with a 1–3 mm margin to deliver the prescribed dose to the tumor. The clinical target volume (CTV) included the GTV and suspected subclinical disease based on clinical risk estimation. A maximum of a 5 mm margin was typically added to the CTV for the planning target volume (PTV) to mitigate potential setup errors and uncertainties in the geometric precision of the dose distribution.

### Dose prescription and delivery

Doses were measured by Gy-equivalents (GyE) to account for the RBE differences of particle RT as compared to photon-based RT. IMPT and IMCT were delivered with PBS technology. For IMPT plan, as we using conventional fractionation,the dose constraints of the OARs were based on the TD5/5 described by Emami et al. [8] However, hypo-perfraction of 2.5–3.0GyE was used in IMCT plan. Due to the lack of experience, extra caution must be applied. The critical organs including the optic nerve (D20 < 30 GyE), brain stem (Dmax< 45 GyE), spinal cord (Dmax< 30 GyE), and temporal lobes (V40 < 7.66 cc; V50 < 4.66 cc) were based on previous experience from the National Institute of Radiation Science (NIRS) of Japan [9]. For patients who were irradiated previously, recovery from previous radiation therapy dose was set at 70% regardless of the latent time between the two courses of RT [10].

### Intensity modulated particle radiation therapy

Treatment planning for IMPT or MICT was performed using the Siemens Syngo® treatment planning system (version VC11). The beam arrangement varied depending on the target volume geometry and dose limits to neighboring organs at risk, such as those with prior radiation exposure. Treatments typically consisted of 2–5 beams with a median of 3 fields. Individual factors such as patient positioning reproducibility and/or beam angles were chosen for optimal dosimetry.

Setup accuracy was confirmed with daily orthogonal X-ray using bony landmarks as a reference. Verification CT scans were typically performed on a weekly basis after the second week of the IMCT course to assess any changes in anatomy. Recalculation was required if the coverage of the CTV is substantially altered, based on the assessment of the attending radiation oncologist.

### Follow-up

All patients were required to adhere to the standardized institutional follow-up protocol. The first follow-up was provided at 4–6 weeks after the completion of particle RT. Patients were then required to be follow-up every 3 months in the first 2 years, every 6 months in the following 3 years, and annually thereafter. A complete H&P examination focused to the head and neck regions, and an enhanced MRI scan of the head and neck area are required at each follow-up. PET-CT, other laboratory, or imaging studies were ordered based on any evidence of metastasis or other concurrent diseases.

### Data analysis

The duration of survival was calculated from the diagnosis of the current condition (first diagnosis or recurrence), until death or the date of the last follow-up. The time to local, regional, distant failure or progression was measured from the date of any treatment for the current diagnosis until documented treatment failure or progression. Freedom from failure and OS rates were calculated using the Kaplan-Meier method. Tumor response was determined as a maximum response on follow-up MRI according to the RECIST criteria. Log-rank test was used for univariate analysis to compare the differences of the survival probabilities. Multivariate analysis using a Cox regression model was performed to define significant prognostic factors. All analyses were performed in SPSS statistics version 24.0 software package (Chicago, IL USA).

Acute adverse events were scored using the Common Terminology Criteria for Adverse Events (CTC AE) version 4.03, and included the adverse events that occurred during or within 3 months after the initiation of particle RT. Late toxicities were scored using the Radiation Therapy Oncology Group (RTOG) late radiation morbidity scoring system for toxicities observed beginning at 90 days after completion of particle RT.

## Results

### Patients and particle RT

Ninety-one consecutive and non-selected patients with cervical and skull base chordomas or chondrosarcomas treated at SPHIC with IMPT or IMCT between 6/2014 and 7/2018 were included in the current analyses. Fourteen (15.4%) patients who had previous photon-based RT (gamma-knife = 7, cyber-knife = 2, or conventional RT = 5) received salvage particle treatment. The remaining 77 patients received particle RT with definitive indication, and most were treated in phase 1 (dose escalation) or phase 2 clinical trials or according to our institutional treatment protocol. The characteristics of patients, their conditions, and treatment were detailed in Table 1.

**Table 1:** Patient characteristics

Parameter		Total *N* = 91(%)
Age (years)/ Median (range)		38(14–76)
Gender	Female	40(44.0)
	Male	51(56.0)
Histology	Chordoma	77(84.6)
	Chondrosarcoma	14(15.4)
Tumor site	Skull base	85(93.4)
	Cervical spinal	6(6.6)
KPS scores	100	26(19.8)
	90	47(51.6)
	80	18(19.8)
GTV/Median (range, cc)		37.0(1.6–231.7)
	≤60 cc	62(68.1)
	>60 cc	29(31.9)
Brainstem compression	No	32(35.2)
	Abutment	14(15.4)
	Yes	45(49.5)
Optic apparatus compression	No	51(56)
	Yes	40(44)
Any compression	No	22(24.2)
	Yes	69(75.8)
Symptom	headache	45(49.5)
	cranial nerve injury	68(74.7)
	endocrine dyscrasia	8(8.8)
Recurrent disease	No	46(50.5)
	Yes	45(49.5)
Re-radiotherapy	No	77(84.6)
	Yes	14(15.4)
Number of surgeries	1	49(53.8)
	2	31(34.1)
	≥3	11(12.1)

Eight patients received IMPT alone to the standard 70 GyE/35 fractions according to the protocol of the registration trial required by the Chinese FDA. The remaining 69 patients were treated according to our institutional dose escalation schemes: 22 patients received IMRT to 50 GyE/25 fractions to CTV 2 plus IMCT boost to the CT-boost to 15~21 GyE/5~8 fractions; 47 patients received 45 GyE/15 fractions to the CTV followed to a boost to CTV-boost to the total of 63~69GyE/21~23 fractions.

For the 14 patients who received re-irradiation, 6 received proton RT (50GyE/25 fractions) followed by IMCT boost to 15~18GyE/5~6 fractions, and 8 received 57~69 GyE in 19~23 fractions. The dose/fraction scheme was decided based on the previous RT regimen and the limitation of the OARs.

### Disease control and survival

**Fig. 1:**
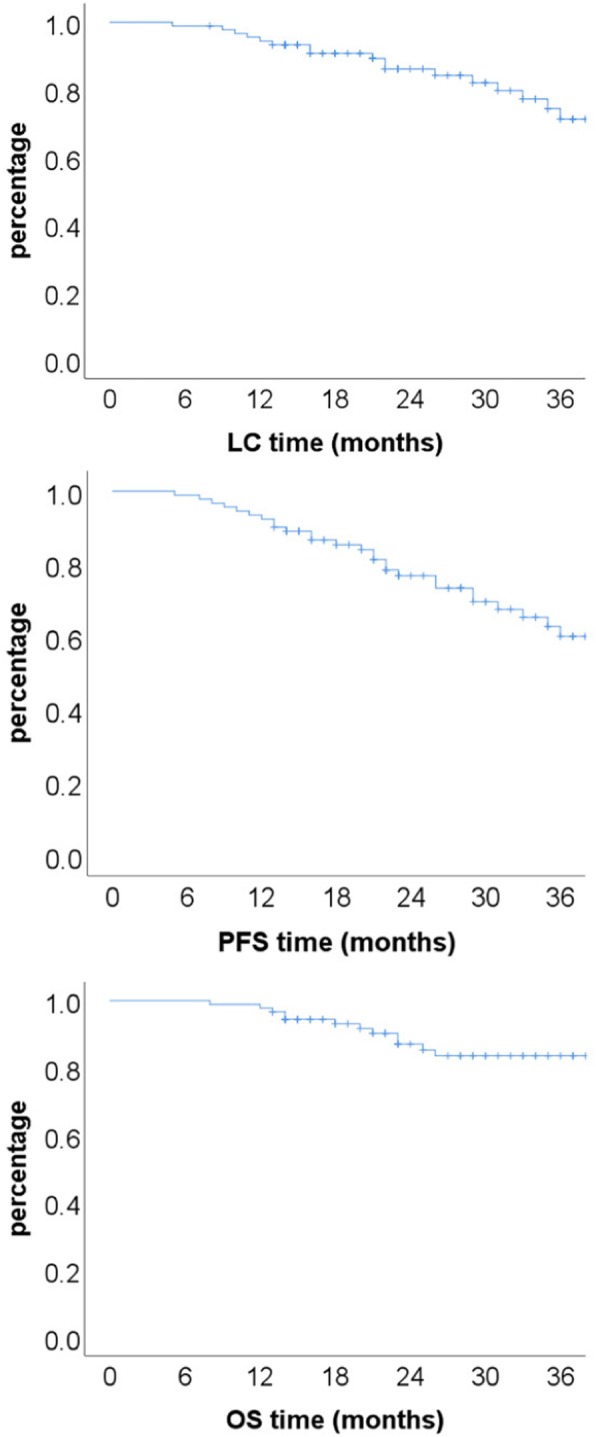
Kaplan Meier survival curves for local control (LC), progression free survival (PFS), and overall survival (OS) rates of the 91 patients with chordoma and chondrosarcoma of the skull base and cervical spine

With a median follow-up time of 28 (range 8–59) months, 28 developed progression/recurrence and 14 of them had deceased. Among the 14 patients deceased at the time of this analysis: 12 (7 re-irradiated and 5 RT-naïve) patients died of uncontrolled local disease including 9 without reaching the RECIST criteria of disease progression, and 2 additional RT-naïve patients died of liver metastasis and stroke, respectively. The 2-year local control, progress free, and overall survival rates were 86.2, 76.8, and 87.2%, respectively for the entire cohort. The 2-year OS rates for patients after first-time radiation vs. re-irradiation were 93.8 50.3%, respectively (p<0.001). Twenty-eight (28) patients developed progression after the completion of particle RT: 7 experienced local recurrence, 8 had local progression, 1 had metastases to cervical spine, 1 had both local progression and metastasis to cervical spine, 1 had liver metastasis, and 9 had uncontrolled local disease (and died) without reaching the RECIST criteria of disease progression. The median time to progression/recurrence was 20.5 (range, 5–48) months. (Fig. 1).

### Prognostic factors

Significant predictive factors for OS and PFS (progression free survival) previously reported in the literature for chordoma and chondrosarcoma were assessed in both uni- and multi-variate analyses. Potential prognostic factors included age, gender, KPS, histology, presence of cranial nerve injury, radiological critical organ compression including brainstem or optic apparatus (with vs. without), GTV volume, as well as technology of particle radiotherapy (IMPT, IMCT, and IMPT + IMCT boost), recurrence status (initial vs. recurrent diagnosis), RT status (RT-naïve vs. re-RT), and surgery stutus (1 vs. > 1 surgery excluding biopsy).

**Fig. 2:**
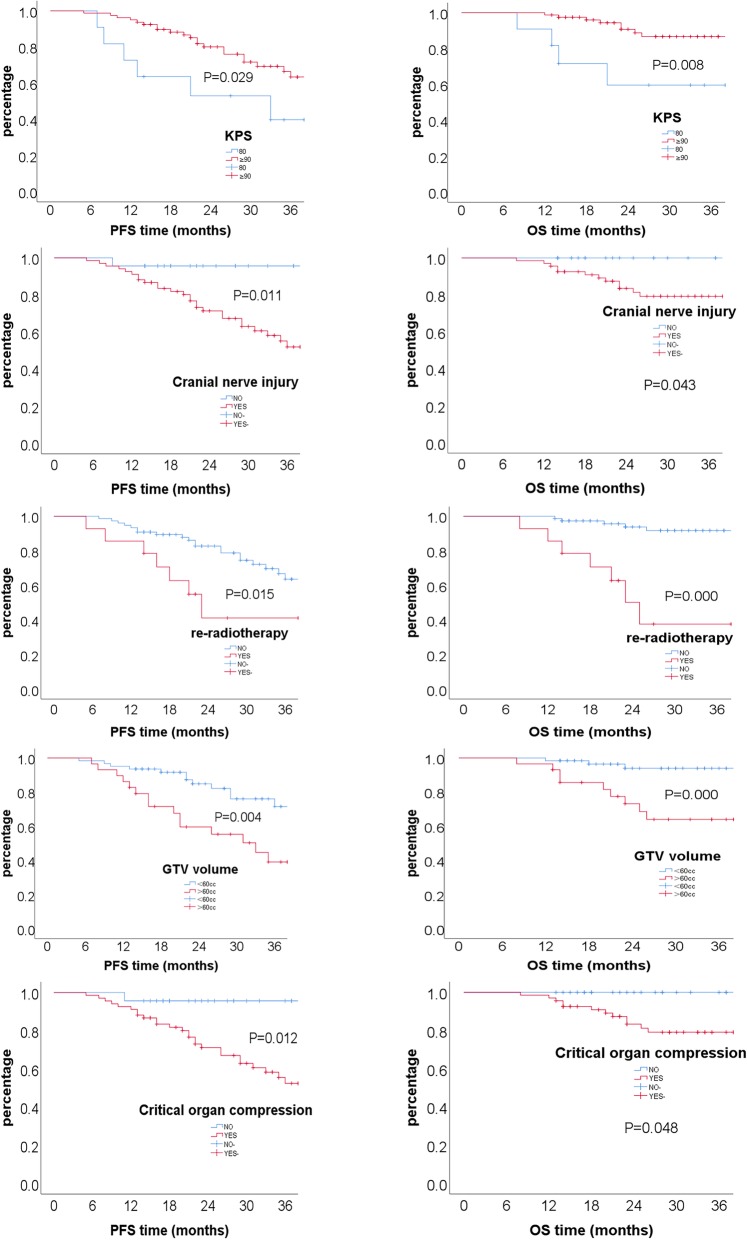
Overall survival (OS) and progression free survival (PFS) for all patients based on Karnofsky performance status (80 vs. ≥90), the presence of cranial nerve injury, nature of radiotherapy (RT vs. re-RT), tumor volume (> vs. < 60 cc), and the presence of critical organ compression

Univariate analysis showed that KPS (*p* = 0.029), the presence of cranial nerve injury (*p* = 0.011), re-irradiation (*p* = 0.015), GTV volume of > 60 cc (*p* = 0.004), as well as brainstem or optic apparatus compression (*p* = 0.012) were significant negative prognosticators for PFS. And KPS (*p* = 0.008), the presence of cranial nerve injury (*p* = 0.043), re-irradiation (p<0.001), GTV volume of > 60 cc (p<0.001), as well as brainstem or optic apparatus compression (*p* = 0.048) were also significant negative prognosticators for OS. (Table 2, Fig. 2).

**Table 2:** Univariate analysis of risk factors for progression free survival (PFS) and overall survival (OS) in 91 patients

Prognostic factors		2-year PFS (%) [95% CI]	*P* value	2-year OS (%) [95% CI]	P value
Age (yeaars)	≤38	64.1(7.5)	0.142	81.8(6.3)	0.130
	>38	89.6(5.0)		92.5(4.3)	
Gender	Female	82.8(6.6)	0.513	88.9(5.3)	0.686
	Male	72.7(6.5)		86.5(5.2)	
KPS	≥90	80.1(4.8)	0.029	90.8(3.6)	0.008
	80	53.0(15.5)		59.7(15.9)	
Critical organ compression	Yes	71.1(5.8)	0.012	83.4(4.9)	0.048
	No	95.7(4.3)		100	
Cranial nerve injury	Yes	71.4(5.8)	0.011	83.5(4.8)	0.043
	No	95.7(4.3)		100	
Histology	Chordoma	73.5(5.5)	0.132	85.9(4.4)	0.340
	Chondrosarcoma	92.9(6.9)		92.9(6.9)	
GTV	≤60 cc	84.9(5.0)	0.004	93.9(3.4)	<0.001
	>60 cc	59.7(9.5)		73.2(8.8)	
Recurrent disease	Yes	73.8(6.9)	0.828	82.7(6.0)	0.260
	No	80.4(6.4)		92.5(4.2)	
Re-radiotherapy	Yes	41.3(15.7)	0.015	50.3(15.5)	<0.001
	No	82.8(4.6)		93.8(3.0)	
Surgery times	1	81.1(6.1)	0.609	87.7(5.2)	0.654
	≥2	71.5(7.3)		86.3(5.7)	
RT	Proton	100	0.69	100	0.489
	Proton+carbon	74.2(8.4)		84.6(7.1)	
	Carbon	75.6(6.3)		87.3(5.1)

On multivariate analyses, GTV volume of > 60 cc (*p* = 0.045) was a significant prognosticator for PFS (Table 4), while GTV volume of > 60 cc (*p* = 0.012) and re-irradiation (*p* = 0.005) were significant prognosticator for OS (Table 3 and Table 4).

**Table 3:** Multivariate analysis of risk factors for progression free survival (PFS)

	B	SE	Wald	df	significance	Exp(B)
KPS	−.031	.058	.273	1	.601	.970
Critical organ compression	1.522	1.058	2.070	1	.150	4.582
Cranial nerve injury	1.312	1.064	1.520	1	.218	3.714
Histology	−.380	.762	.249	1	.618	.684
Tumor volume	1.037	.517	4.030	1	.045	2.822
Recurrent disease	−.927	.631	2.162	1	.141	.396
Re-radiotherapy	.774	.553	1.956	1	.162	2.168
Surgery	−.076	.574	.018	1	.895	.927

**Table 4:** Multivariate analysis of risk factors for overall survival (OS)

	B	SE	Wald	df	significance	Exp(B)
KPS	.003	.077	.002	1	.967	1.003
Critical organ compression	11.162	195.729	.003	1	.955	70,401.455
Cranial nerve injury	11.376	321.728	.001	1	.972	87,178.423
Histology	.500	1.105	.205	1	.651	1.649
Tumor volume	2.081	.824	6.372	1	.012	8.013
Recurrent disease	−1.116	1.034	1.166	1	.280	.327
Re-radiotherapy	2.376	.840	7.993	1	.005	10.759
Surgery	−.896	.811	1.220	1	.269	.408

### Adverse effects

A total of 25 (27.5%) patients developed Grade 1–2 oral mucositis, including 10 (11.9%) Grade 2 mucositis. Only one patient developed Grade 3 acute mucositis. Eight (20.2%) patients experienced grade 1 skin reaction and 9 (9.9%) with focal hair loss. Other acute toxicities included grade 1 myelosuppression in 6 patients, and grade 1 hearing loss in 5 patients.

Late toxicities of grade 1 or 2 were observed in 19 patients: 9 with hearing loss, 5 with xerostomia, 1 with dysphagia, another with reduced prolactin, and 3 with unilateral white matter changes in the temporal lobe (1 also experienced memory loss). No patient experienced severe toxicities of grade 3 or above.

## Discussion

The present study reported the first 91 consecutive and non-selected patients with skull base or cervical spine chordoma and chondrosarcoma patients treated with proton and/or carbon ion therapy at the Shanghai Proton and Heavy Ion Center. A substantial portion of patients presented with locally recurrent disease after surgery or surgery followed by photon radiotherapy. We have discovered that the 2-year os for radiation naïve patients was 93.8%, significantly better than 50.3% of those who failed prior photon radiation. Furthermore, tumor volume (> vs. < 60 cc) was a significant prognosticator for PFS, and re-irradiation and tumor volume were significant prognosticators for os.

Chordomas and chondrosarcomas are rare malignancies that present multiple management challenges because of their proximity to vital structures and relative resistance to chemotherapy and radiotherapy. Both conditions are highly locally invasive and usually occur near critical organs especially the central nervous system (CNS), thus those occurs in the head and neck are often amalgamated because of similar clinical presentation, radiologic finding, anatomic location, and treatment. Maximal safe resection combined with adjuvant RT is a key component of successful management of cervical and skull base chordoma and chondrosarcoma. However, even by experienced teams, the complete resection rate in published studies has ranged from 0 to 60%. In a study of 159 skull base chondrosarcoma patients reported by Feuvret et al. [11], the complete resection rate was only 8.2%. Gay et al. [12] attempted radical surgery for this condition. However, only 60% could undergo surgery and 30% experienced cerebrospinal fluid leakage. Therefore, adjuvant RT is considered necessary for most if not all patients, and could improve local control especially for patients with residual disease after surgery [3].

Because of their radio-resistance, high doses are needed for successful local control for chordomas and chondrosarcomas. However, the doses/fractions at 70–74 GyE (1.8-2GyE) or 60.8–67.6 GyE (hypo-fractionation at 3–4.2 GyE) that is considered minimum efficacious doses for local control are difficult to achieve with photon-based RT [6, 13, 14]. Particle RT is featured with its far superior physical properties and enables more focused dose delivery to the tumor volumes thereby improves therapeutic ratio especially for tumors positioned near critical OARs. In addition, carbon-ion beam has higher linear-energy transfer (LET) and may inflict higher relative biological effectiveness [5]. As such, proton or carbon-ion RT is more superior RT technique for skull base tumors that requires high-dose irradiation. The outcomes after proton RT had been reported in numerous retrospective studies. The 5-year OS rates ranged between 67%~ 81%, and those of LC rates ranged between 46%~ 81% [15]. Schulz-Ertner et al. of the Heidelberg Ion Therapy Center (HIT) reported their experience of skull base chordomas using the median carbon ion dose of 60 GyE in 20 fractions [16, 17]. The 3-year OS and LC rates were 80.6 and 91.8%, respectively. In a phase I/II dose escalation study from the NIRS of Japan with a total dose of 48.0, 52.8, 57.6, and 60.8 GyE in 16 fractions, Mizoe et al. reported that 60.8 GyE/16 fractions of carbon ion radiotherapy provided the best local control (at 100%) at 5 years [18]. Cautions must be applied when comparing the outcomes using different beam types or carbon ion therapy using different biological models. In the current study, we analyzed our patients treated at different dosage schemes of proton and or carbon ion therapy mostly according to our trial protocols. Findings at 2-years for RT-naïve patients were similar to data reported in previous literatures: the OS and LPFS rates were ~ 94% and ~ 83%, respectively, which are significantly better than those after salvage particle radiotherapy. In addition, we didn’t find any significant differences between any kinds of particle therapy or the dosage levels for local control or overall survival due to, at least in part, relatively high RBE doses used and relatively short follow-up.

The size of GTV is the most frequently reported prognostic factor for local control or OS in patients with skull base chordomas and chondrosarcomas [6, 19]. McDonald et al. reported postoperative GTV had a statistically significant association with LC on multivariate analysis, whereas preoperative GTV had no apparent association with outcomes [20]. This supports a strategy of maximal resection prior to adjuvant RT. Unfortunately, the extent of surgery is usually limited by risk of severe neurological morbidities. GTV with cut points from 20 to 110 cc were used to report its association with the outcomes [4, 6, 19, 20] [[21, 22], and GTV > 25 mL was mostly reported as the negative factor for local control [4, 6]. As the majority of patients in our series presented with locally advanced diseases, a higher tumor volume (i.e., > 60 CC) was found to be the cut off for predicting both OS and PFS. The high proportion of patients with more advanced disease in our cohort could be the reason, at least in part, for the slightly inferior LC rate in our study. For tumors with large volumes, especially when compression of CNS is evident, a portion of the tumor may be under dosed in order to meet constraints to critical OARs such as optic nerves or brainstem. Optic apparatus or brainstem compression was identified as a prognostic factor for patient’s outcome in the univariate analyses of our study. Our finding echoed the series previously reported. Weber et al. [23] reported the 7-year OS rates of 73.7 and 90.3% for patient with or without optic apparatus or brainstem compression, respectively (*P* = 0.025). Hug et al. [4] reported the local control rate for patients with or without brainstem involvement were 53 and 94%, respectively (*p* = 0.04). Our results also demonstrated that the presence of cranial nerve injury was a significant negative prognosticator in the univariate analysis. However, compression of the critical structures of the central nervous system was not shown to be significant in our multi-various as significant factors, because most of the compressions were presented in patients with larger GTV.

One feature of our study was that close to 50% of patients presented with recurrent disease after surgery with or without prior adjuvant photon RT. Patients with locally recurrent chordoma and chondrosarcoma represent a significant clinical challenge, with few promising treatment options and few articles reported the results of these disease [24]. Surgical re-resection provides a transient benefit only for most patients. Fagundes et al. reported on 49 patients who received salvage therapy for local relapse of chordomas of the base of skull or cervical spine [25]. The 2-year OS rates after salvage therapy was 63%. In our study, 45 of the 91 patients (49.5%) presented as recurrent tumors, 44 (46.2%) had 2 or more surgeries, and 14 (15.4%) failed previous photon-based RT. In addition, all patients received R2 resection. The 2-year OS was 80.7% for this sub-group of patients. Although we didn’t find significant differences in PFS and OS between recurrent and primary tumors, patients who failed previous course of RT had significantly worse outcomes. Similar findings were demonstrated in the experience from M.D. Anderson Cancer Center. Holliday et al. reported that in 19 patients with spinal chordomas and chondrosarcomas, those who received proton RT at initial presentation had a local control rate of 80%, as compared with 46% in those who were treated at the time of recurrence [26]. Indelicato et al. [27] reported the similar results of 51 patients with spinal chordoma, and the 4-year LC rate after initial radiotherapy was 71% versus 19% for those irradiated for recurrence. McDonald et al. [28] reported 16 chordoma patients treated with a median re-irradiation dose of 75.6 GyE proton therapy, and the 2-year OS rate was 80%. However, higher dosage of re-irradiation led to more toxicity, and the 2-year estimate grade 3 and 4 late toxicity was as high as 19%. Re-irradiation would certainly increase the risk to OARs for radiation-induced toxicities. The concerns of overdosing brainstem, spinal cord, or optic apparatus constrained the doses of second course of RT. It is difficult to derive clear guidelines for normal tissue dose constraints in the setting of re-irradiation, given the numerous factors involved that may influence organ tolerance.

Although we presented the largest series of skull base/cervical chordoma and chondrosarcoma treated mainly with IMCT component, and most patients were treated in a prospective manor, a number of pitfalls need to be discussed. First, the patient composition of our series is relatively heterogenic, and recurrent disease consisted close to 50% of patients. Treatments received prior to our proton and carbon-ion RT were also mixed. Second, the follow-up time of 2 years for the entire cohort was short, although such a time frame maybe sufficient for recurrent diseases received salvage treatment for short-term outcomes. Further follow-up is surely necessary to assess the durability of disease control and to monitor for additional particle RT-induced late toxicities. In addition, chordoma seemed to have a suboptimal outcome compared to chondrosarcoma in reported literatures [6, 23]; however, there was no significant survival difference in our study due to, at least in part, the limited patient number for chondrosarcomas and short follow-up time. Third, most of our patients were treated in prospective phase 1 and 2 trials. For those received dose-escalating schemes, different dose and combination of beams were used. Nevertheless, the biological equivalent doses are considered sufficient even at the lower levels in phase 1 trials based on historical data.

## Conclusions

Proton and/or carbon ion therapy was effective and safe for patients with skull base or cervical spine chordoma and chondrosarcoma. Larger tumor volume (> 60 cc) and re-irradiation were significant prognostic factors and predicts inferior survival. Further follow-up is needed for long-term efficacy and safety.

## Data Availability

The datasets used and analyzed during the current study are available from the corresponding author on reasonable request.

## Abbreviations

CNS: central nervous system
CTC AE: the Common Terminology Criteria for Adverse Events
CTV: clinical target volume
GTV: gross tumor volume
HIT: Heidelberg Ion Therapy Center
IMCT: intensity-modulated carbon ion radiotherapy
IMPT: intensity-modulated proton therapy
LC: local control
LET: linear-energy transfer
MRI: magnetic resonance imaging
OARs: organs at risk
OS: overall survival
PBS: pencil beam scanning
PET-CT: positron emission tomography
PFS: progression free survival
PTV: planning target volume
RBE: relative biological effectiveness
RTOG: the Radiation Therapy Oncology Group
SPHIC: Shanghai Proton and Heavy Ion Center
